# IL-24 Is a Promising Molecular Adjuvant for Enhancing Protective Immunity Induced by DNA Vaccination Against *Toxoplasma gondii*

**DOI:** 10.3390/microorganisms13071661

**Published:** 2025-07-14

**Authors:** Bohuai Xu, Xiuqiang Zhang, Yaowen Wang, Jia Chen

**Affiliations:** 1 The First Affiliated Hospital of Ningbo University, Ningbo 315000, China; fyxubohuai@nbu.edu.cn (B.X.); zhangxiuqiang75@163.com (X.Z.); wangyaowennihao@hotmail.com (Y.W.); 2 Health Science Center, Ningbo University, Ningbo 315211, China

**Keywords:** protozoan vaccine, serological markers, Th1 immunity, T-cell activation, protective efficacy

## Abstract

*Toxoplasma gondii*, a parasitic protozoan, causes zoonotic infections with severe health impacts in humans and warm-blooded animals, underscoring the urgent need for effective vaccines to control these infections. In this study, a DNA vaccine encoding TgROP5, TgROP18, TgGRA7, TgGRA15, and TgMIC6 was formulated using the eukaryotic expression vector pVAX I. IL-24 was delivered as a molecular adjuvant using plasmid pVAX-IL-24. BALB/c, C57BL/6, and Kunming mouse strains received the DNA immunization, after which antibody levels, cytokine production, and lymphocyte surface markers were analyzed to assess immune responses. Additionally, survival rates and brain cyst counts were measured 1 to 2 months post-vaccination in experimental models of toxoplasmosis. As a result, compared to controls, the DNA vaccine cocktail significantly increased serum IgG levels, Th1 cytokine production, and proportions of CD4+/CD8+ T cells, leading to extended survival and reduced brain cyst counts post-challenge with *T. gondii* ME49. Furthermore, the five-gene DNA vaccine cocktail conferred greater protection compared to single-gene immunizations. Co-administration of IL-24 significantly enhanced the immune efficacy of the multi-gene DNA vaccination. Our findings suggest that IL-24 is an effective molecular adjuvant, enhancing the protective immunity of DNA vaccines against *T. gondii*, supporting its potential role in vaccine strategies targeting other apicomplexan parasites.

## 1. Introduction

*Toxoplasma gondii* is an intracellular protozoan that infects a wide range of warm-blooded animals, including humans, causing toxoplasmosis. While infection is often asymptomatic in immunocompetent individuals, it poses severe risks to immunocompromised patients and pregnant women, potentially leading to life-threatening complications or congenital defects. In livestock, *T. gondii* infection can cause abortion and neonatal loss, resulting in significant economic burdens [1,2]. Current treatments are ineffective against latent tissue cysts, and concerns over drug resistance highlight the urgent need for alternative preventive strategies [3].

Vaccination is a promising approach to controlling *T. gondii* infections, but existing options are limited. The only licensed vaccine, the live-attenuated S48 strain, is restricted to sheep due to safety concerns, making it unsuitable for broader applications [4]. DNA vaccines have emerged as a viable alternative, offering advantages in safety and immunogenicity. Among potential antigen candidates, rhoptry proteins (ROP5, ROP18), dense granule proteins (GRA7, GRA15), and microneme proteins (MIC6) have shown protective efficacy in mouse models [5,6]. However, single-gene DNA vaccines often fail to elicit robust immunity, suggesting that a multi-antigen approach may provide superior protection [7].

Genetic adjuvants, including cytokines, have been explored to enhance vaccine-induced immune responses. While cytokines such as IL-33 and IL-15 have demonstrated potential, toxicity concerns limit their practical application [8,9]. IL-24, a member of the IL-10 family, plays a role in immunoregulation and has shown promise in enhancing T cell-mediated immunity with low toxicity [10]. However, its potential as a molecular adjuvant in DNA vaccines against infectious diseases, including toxoplasmosis, remains unexplored.

This study evaluates the immunogenicity and protective efficacy of a multi-antigen DNA vaccine incorporating *T. gondii* ROP5, ROP18, GRA7, GRA15, and MIC6. Additionally, we investigate the role of IL-24 as a genetic adjuvant to determine whether it enhances vaccine-induced immunity against acute and chronic *T. gondii* infections in different mouse strains. Our findings may provide insights into the development of more effective vaccine strategies for *T. gondii* and related pathogens.

## 2. Materials and Methods

### 2.1. Mice

Six- to eight-week-old specific pathogen-free female Kunming outbred mice, inbred BALB/c mice, and C57BL/6 mice were purchased from the Zhejiang Experimental Animal Center in Hangzhou, China. All mice were maintained in accordance with the Animal Ethics Procedures and Guidelines of the China. The study received approval from the ethical committee of Ningbo University [permission: AEWC-NBU20230274; 3 April 2023].

### 2.2. Parasites, Cells, and Antigens

Tachyzoites of the *T. gondii* RH strain (Type I) and cysts of the ME49 strain (Type II) were propagated, harvested, and used for the in vivo challenge of mice, as described previously [11,12]. The obtained tachyzoites were utilized for the preparation of *T. gondii* lysate antigen (TLA) as previously detailed [12]. Human Embryonic Kidney 293T (HEK 293-T) cells were used for transfection and were grown in Dulbecco’s Modified Eagle’s Medium (DMEM; Invitrogen) supplemented with 10% (*v*/*v*) heat-inactivated fetal calf serum (FCS), 100 IU/mL streptomycin, and 100 IU/mL penicillin at 37 °C in a 5% CO_2_ atmosphere.

### 2.3. Construction of the Eukaryotic Expression Plasmid

To construct the pVAX I plasmid encoding IL-24, we used reverse transcription-PCR (RT-PCR) to amplify total RNA isolated from the spleens of Kunming mice, following previously established methods [13]. A pair of oligonucleotide primers was used (forward primer: 5′-GGGGTACC ATGCGATCGGATCCAGCTAAT-3′, reverse primer: 5′-GCTCTAGA CACATGCCTCATAGTCGCAG-3′), which introduced Kpn I and Xba I restriction sites. The resulting PCR product was inserted into the pMD-18 T Vector (TaKaRa, Dalian, China), generating pMD-IL-24. This plasmid was subsequently cleaved with Kpn I and Xba I, then subcloned into the pVAX I vector (Invitrogen), also cleaved with the same enzymes, using T4 DNA ligase to generate the pVAX-IL-24 plasmid. The recombinant plasmids were verified through PCR, double restriction enzyme digestion, and sequencing.

The pVAX I plasmids expressing TgROP5, TgROP18, TgGRA7, TgGRA15, and TgMIC6 were constructed according to our previously described [9,14,15], with the fidelity of all plasmids confirmed by double enzyme digestion and sequencing (Sangon, Shanghai, China). The positive plasmids were purified from transformed *Escherichia coli* DH5α cells using anion exchange chromatography (EndoFree plasmid giga kit, Qiagen Sciences, Germantown MD, USA) according to the manufacturer’s instructions. The concentration and purity of the plasmids were assessed using a spectrophotometer, measuring optical densities at 260 and 280 nm (OD260 and OD280). The purified plasmids were stored at −20 °C until needed for mouse immunization protocols.

### 2.4. Expression of pVAX-IL-24 Plasmid In Vitro

To confirm the expression of pVAX-IL-24 in vitro, HEK 293-T cells were transfected with pVAX-IL-24 or an empty vector (control plasmid) using Lipofectamine™ 2000 reagent (Invitrogen, Carlsbad, CA, USA) according to the manufacturer’s instructions. Forty-eight hours post-transfection, ELISA kits were employed to determine the concentration of IL-24 in the supernatants of the transfected cells, following the manufacturer’s guidelines (Mouse IL-24 ELISA Kit, Abcam, Cambridge, UK), as previously described [13].

### 2.5. DNA Immunization and Challenge Infection

For each mouse strain, a total of 320 mice were randomly divided into 11 groups of 29 mice each. The vaccination regimens for each group are detailed (the vaccination procedure is identical in each mouse strain) in Table 1. Mice were immunized three times at two-week intervals (weeks 0, 2, and 4) by intramuscular injection of 100 μg of plasmid DNA in 100 μL of sterile PBS into the tibialis anterior muscle, using a 1 mL insulin syringe with a 28-G needle. Two control groups received either 100 μg of the empty pVAX vector or PBS (100 μL each), while one group of mice remained uninoculated to serve as a blank control. Blood was collected from the tail vein prior to each immunization and challenge infection, and sera were separated and stored at −20 °C until analyzed for specific antibodies.

**Figure 1:**
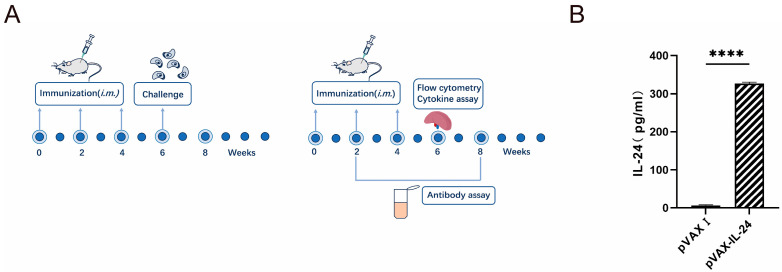
Determination of the expression of pVAX-IL-24 in vitro in 293-T cells by ELISA and immunization. (**A**) Flow chart of mice immunization and immunological analyses. (**B**) 293-T cells were transfected with empty pVAX I or pVAX-IL-24. Statistical significance is indicated as **** *p* < 0.0001.

For the challenge in each mouse strain, 8 mice were intraperitoneally inoculated with 1 × 10^3^ tachyzoites of the virulent *T. gondii* RH strain, and another 8 mice were orally challenged with 100 cysts of the *T. gondii* ME49 strain, with mortality recorded until all animals succumbed. An additional 6 mice were orally challenged with 10 cysts of the *T. gondii* ME49 strain 14 days after the last immunization, and cysts in their brains were counted 30 days post-challenge. Two weeks after the final immunization, nine mice per group were sacrificed to harvest splenocytes for flow cytometric analysis (three mice), lymphoproliferation assays (three mice), and cytokine measurements (three mice). The entire vaccine preparation process is illustrated in the flowchart in Figure 1A.

### 2.6. Measurement of Humoral Immune Responses

Enzyme-linked immunosorbent assay (ELISA) was employed to detect IgG, IgG1, and IgG2a antibodies in serum samples collected at weeks 0, 2, 4, and 6. The assays were conducted using the SBA Clonotyping System-HRP Kit (Southern Biotech Co., Ltd., Birmingham, UK), following previously described methods [11]. Briefly, 100 µL of TLA (10 µg/mL) in PBS was added to each well and incubated overnight at 4 °C. After washing three times with PBST, the plates were blocked with 1× PBS containing 1% BSA at 37 °C for 1 h. Serum samples diluted in PBS (1:100) were then added and incubated at room temperature for 1 h. After washing with PBST, 100 μL of HRP-conjugated anti-mouse IgG, IgG1, and IgG2a were added to each well and incubated at 37 °C for 60 min. Following another wash with PBST, binding was visualized by incubating with 100 μL of substrate solution (1.05% citrate substrate buffer, 1.5% ABTS, 0.03% H_2_O_2_, pH 4.0) for 30 min. Absorbance was measured at 450 nm using an ELISA reader (Bio-Tek EL × 800, Winooski, VT, USA). All experimental and control samples were run in triplicate.

### 2.7. Lymphocyte Proliferation Assays

Two weeks after the third DNA immunization, splenocytes were aseptically harvested from three mice in each group as previously described [11,12]. Erythrocytes were lysed using erythrocyte lysis buffer (Sigma, St. Louis, MO, USA). After washing with PBS, the splenocytes were resuspended in DMEM supplemented with 10% FCS. Subsequently, 2 × 10^5^ cells per well were cultured in 96-well Costar plates with TLA (10 μg/mL), concanavalin A (ConA; 5 μg/mL; Sigma) as a positive control, or medium alone as a negative control, at 37 °C under a 5% CO_2_ atmosphere for 72 h. Following this incubation, 10 µL of 3-(4,5-dimethylthiazol-2-yl)-2,5-diphenyltetrazolium bromide (MTT; 5 mg/mL, Sigma) was added to each well and incubated for an additional 4 h. The stimulation index (SI) was calculated using the formula: SI = OD_570_ (ConA)/OD_570_ (medium). All experimental and control samples were run in triplicate.

### 2.8. Cytokine Assays

Splenocytes were harvested as described for the lymphocyte proliferation assay, and different stimuli (TLA, ConA for positive control; medium alone for negative control) were added to corresponding wells in flat-bottom 96-well microtiter plates. Culture supernatants were collected and analyzed for IFN-γ at 96 h and for IL-2, IL-4, and IL-10 at 24 and 72 h, respectively, following protocols previously reported [12,13]. Cytokine concentrations were determined using commercial ELISA kits (Biolegend, San Diego, CA, USA) based on standard curves generated from known amounts of mouse recombinant IFN-γ, IL-2, IL-4, and IL-10. The sensitivity limits for the assays were 8.0 pg/mL for IFN-γ, 0.9 pg/mL for IL-2, 0.5 pg/mL for IL-4, and 23.8 pg/mL for IL-10. Data analysis was performed using results from three independent experiments.

### 2.9. Flow Cytometry Analysis

As previously described [11,12], the percentages of CD4+ and CD8+ T lymphocytes were analyzed by flow cytometry. In brief, splenocyte suspensions were stained with phycoerythrin-labeled anti-mouse CD3 (eBioscience, San Diego, CA, USA), allophycocyanin-labeled anti-mouse CD4 (eBioscience), and fluorescein isothiocyanate-labeled anti-mouse CD8 (eBioscience) antibodies at 4 °C for 30 min in the dark. The samples were then fixed with FACScan buffer (PBS containing 1% BSA and 0.1% sodium azide) and 2% paraformaldehyde. Fluorescence profiles were analyzed using a FACScan flow cytometer (BD Biosciences) with SYSTEM II software v2.4 (Coulter, Brea, CA, USA).

### 2.10. Statistical Analysis

Statistical analyses were performed using SPSS 13.0 Data Editor (SPSS Inc., Chicago, IL, USA). Differences in data (e.g., antibody responses, lymphoproliferation assays, and cytokine production) between groups were compared using Student’s *t*-test. The standard error was calculated using the “stdevp” function in Microsoft Excel. Results were considered statistically significant if *p* < 0.05.

## 3. Results

### 3.1. Identification of Plasmids

To confirm the positive plasmids, five purified constructs—pVAX-ROP5, pVAX-ROP18, pVAX-GRA7, pVAX-GRA15, and pVAX-MIC6—were sequenced and analyzed for alignment. Sequence alignment with corresponding entries in GenBank (accession numbers MW521219.1, GQ243216.1, XM_0023771901.2, DQ459451.2, and AF110270.1) revealed no base deletions or alterations. To assess the expression of pVAX-IL-24 in vitro, IL-24 levels were measured by ELISA following transfection into HEK 293-T cells. The measurement range for IL-24 was 40 pg/mL to 2000 pg/mL. As shown in Figure 1B, the results indicated that transfection with pVAX-IL-24 resulted in a high IL-24 concentration in the supernatants of HEK 293-T cells, while no IL-24 was detected in cells transfected with the empty pVAX I vector.

### 3.2. Humoral Immune Responses

**Figure 2:**
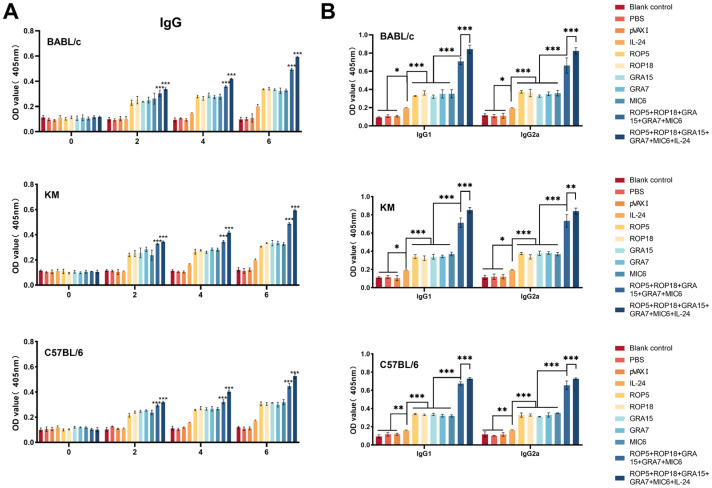
Detection of specific anti-*T. gondii* humoral immune responses induced by DNA immunization with single or multiple genes in different mouse strains. (**A**) Measurement of IgG antibodies in the sera of Kunming mice at 0, 2, 4, and 6 weeks post-immunization. (**B**) Quantification of IgG1 and IgG2a antibodies in immunized mice two weeks after the final vaccination. Statistical significance is indicated as *** *p* < 0.001, ** *p* < 0.01, * *p* < 0.05. Data are presented as means ± SD.

The total IgG and subclasses (IgG1 and IgG2a) in serum samples from immunized and control groups were evaluated using standard ELISA after three consecutive DNA immunizations (weeks 0, 2, 4, and 6). As shown in Figure 2A, the IgG levels were significantly higher (*p* < 0.05) in the group receiving the combination of pVAX-IL-24, pVAX-ROP5, pVAX-ROP18, pVAX-GRA7, pVAX-GRA15, and pVAX-MIC6 compared to other immunized groups. Moreover, the group immunized with pVAX-ROP5, pVAX-ROP18, pVAX-GRA7, pVAX-GRA15, and pVAX-MIC6 exhibited significantly elevated IgG levels compared to those immunized with individual plasmids. Additionally, pVAX-IL-24 elicited a notable IgG response compared to control groups. Notably, IgG levels increased in all immunized groups over time, peaking four weeks after the final immunization. In contrast, no significant increase in antibody levels was observed among the three control groups (*p* > 0.05).

As shown in Figure 2B, levels of IgG1 and IgG2a, as well as the IgG2a/IgG1 ratio, were markedly higher in the immunized groups compared to the control groups. Similar to the IgG levels, the IgG2a/IgG1 ratios were significantly higher in the group receiving the combination of pVAX-ROP5, pVAX-ROP18, pVAX-GRA7, pVAX-GRA15, and pVAX-MIC6 compared to those immunized with single plasmids. Co-administration of pVAX-IL-24 with the multi-gene vaccine resulted in the highest IgG2a/IgG1 ratio; however, no significant differences in the IgG2a/IgG1 ratio were observed among the control groups (*p* > 0.05). In terms of mouse strains, IgG and subclass (IgG1 and IgG2a) levels were comparable between vaccinated BALB/c and Kunming mice, while both vaccinated and control C57BL/6 mice exhibited lower IgG and subclass levels (Figure 2A,B).

### 3.3. Cellular Immune Responses

**Figure 3:**
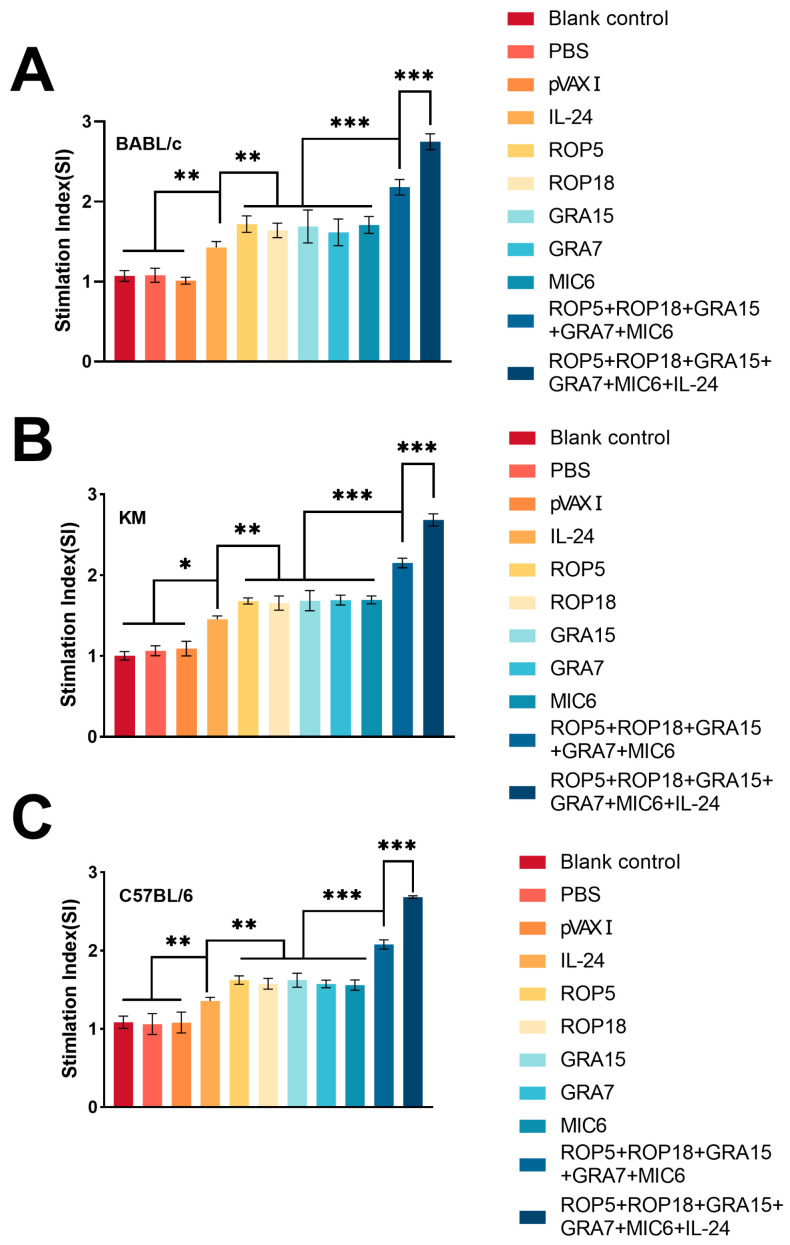
Splenocyte proliferative response in immunized and control mice across different mouse strains. (**A**) Stimulation index (SI) for lymphocyte proliferation in immunized and control BALB/c mice, *n* = 3/group. (**B**) Stimulation index (SI) for lymphocyte proliferation in immunized and control Kunming mice, *n* = 3/group. (**C**) Stimulation index (SI) for lymphocyte proliferation in immunized and control C57BL/6 mice, *n* = 3/group. Statistical significance is indicated as *** *p* < 0.001, ** *p* < 0.01, * *p* < 0.05. Data are presented as means ± SD.

The lymphocyte proliferative response was evaluated following stimulation with TLA or ConA using the MTT assay. As shown in Figure 3, the stimulation index (SI) in spleen cells from all vaccinated groups was higher than that of non-immunized controls. The administration of pVAX-IL-24 significantly enhanced the SI for the multi-gene DNA vaccine plasmids; however, no significant differences in SI were found among the groups receiving single-plasmid immunization (*p* > 0.05). Similar proliferative responses were observed in spleen cells from vaccinated BALB/c (Figure 3A), Kunming mice (Figure 3B), and C57BL/6 (Figure 3C) following stimulation with TLA or ConA.

**Figure 4:**
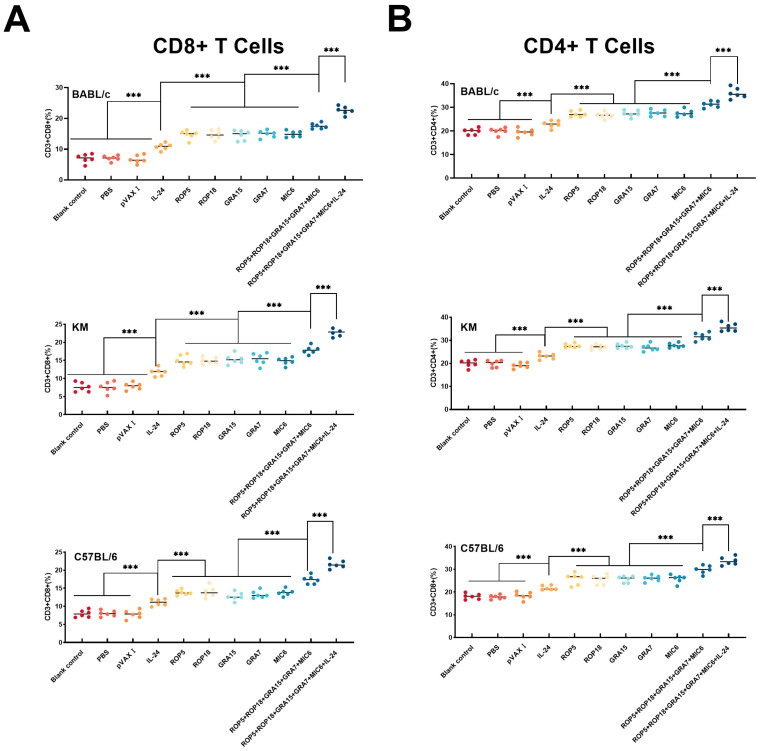
Percentages of CD4+ and CD8+ T cells in immunized and control mice across different mouse strains. (**A**) The proportion of CD4+ T cells is shown for both immunized and control groups in BALB/c, C57BL/6, and Kunming mice, *n* = 3/group in each mouse strain. (**B**) The proportion of CD8+ T cells is shown for both immunized and control groups in BALB/c, C57BL/6, and Kunming mice, *n* = 3/group in each mouse strain. Statistical significance is indicated as *** *p* < 0.001. Data are presented as means ± SD.

To further characterize the cellular immune response, flow cytometry was utilized to determine the percentages of CD3+ CD4+ and CD3+ CD8+ T cell subsets in the spleens of mice from each group. As demonstrated in Figure 4, the percentages of CD3+ CD4+ and CD3+ CD8+ T lymphocyte subsets were significantly higher in the experimental groups compared to the controls (blank, PBS, pVAX I). Additionally, a considerably higher percentage of CD4+ T cells (Figure 4A) and CD8+ T cells (Figure 4B) was observed in the groups receiving the cocktail of DNA vaccines versus those receiving a single-gene plasmid (*p* > 0.05). Furthermore, the co-administration of pVAX-IL-24 enhanced the cellular immune response induced by multiple-gene DNA immunization (*p* < 0.05). Similar to the proliferative responses, there were no significant differences in cellular immune responses among the different mouse strains.

### 3.4. Cytokine Production

Splenocytes from both immunized and non-immunized mice were harvested two weeks after the last immunization, and cytokine levels were evaluated by ELISA following stimulation with TLA. Consistent with earlier results, multiple-gene DNA immunization induced higher cytokine levels than those seen in the groups immunized with a single-gene plasmid. The highest cytokine levels were observed in the groups immunized with pVAX-IL-24 in combination with the multi-gene DNA vaccines. However, no significant differences were noted among the three control groups (*p* > 0.05).

**Figure 5:**
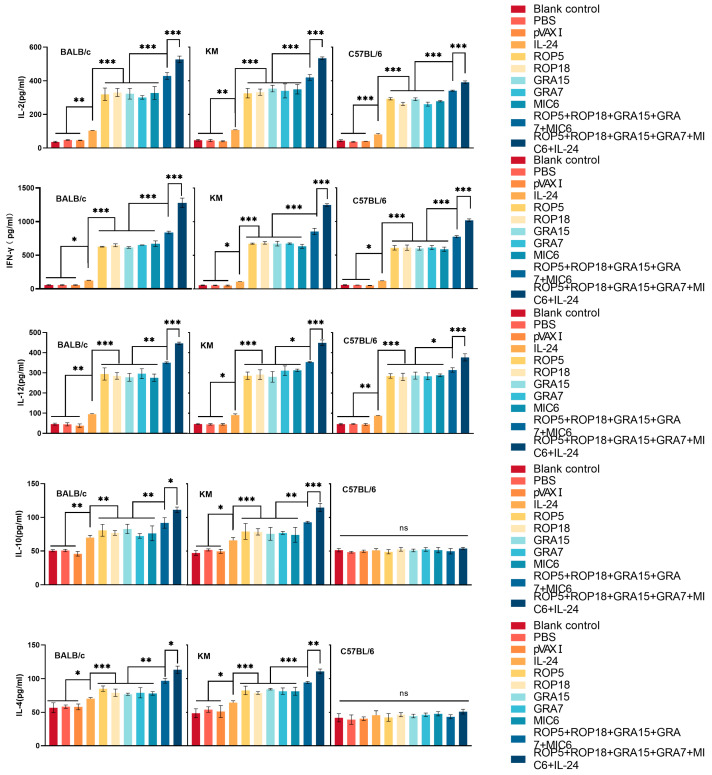
Cytokine production by splenocytes from mice immunized with single or multiple genes across different mouse strains. Statistical significance is indicated as *** *p* < 0.001, ** *p* < 0.01, * *p* < 0.05, and ns: no significance. Data are presented as means ± SD.

Regarding Th1-associated cytokines, significantly elevated levels of IFN-γ, IL-2, and IL-12 were detected in splenocyte cultures from all immunized Kunming, C57BL/6, and BALB/c mice compared to controls. Among the mouse strains, splenocytes from immunized Kunming and BALB/c mice produced significantly higher levels of IFN-γ, IL-2, and IL-12 than those from vaccinated C57BL/6 mice (Figure 5). Additionally, splenocytes from DNA-vaccinated BALB/c and Kunming mice secreted the Th2-associated cytokines IL-4 and IL-10 upon stimulation with TLA, while production of both IL-4 and IL-10 was undetectable in splenocyte supernatants from both vaccinated and control C57BL/6 mice.

### 3.5. Assessment of Protective Activity

**Figure 6:**
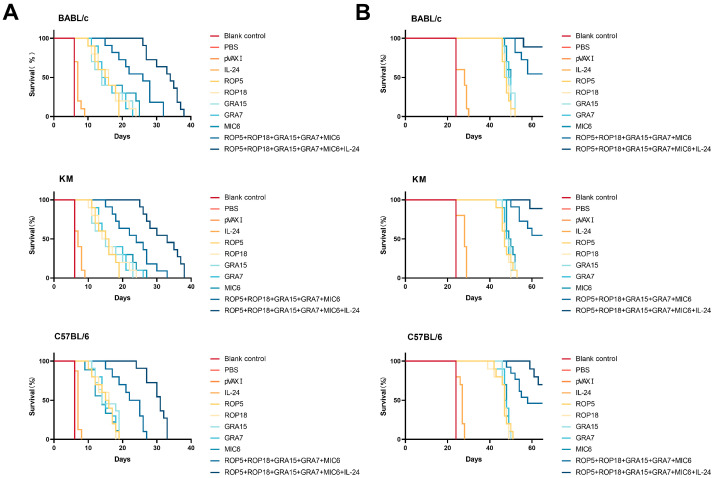
Survival curves of immunized BALB/c, C57BL/6, and Kunming mice two weeks after the final immunization. (**A**) Survival rates of immunized mice (BALB/c, C57BL/6, and Kunming mice) challenged with 1 × 10^3^ tachyzoites of the RH strain, *n* = 8/group in each mouse strain. (**B**) Survival rates of immunized mice (BALB/c, C57BL/6, and Kunming mice) challenged with 100 cysts of the ME49 strain, *n* = 8/group in each mouse strain 100 cysts of the ME49 strain.

Following challenges with a lethal dose of 100 tissue cysts from the *T. gondii* ME49 strain or 1 × 10^3^ tachyzoites of the virulent RH strain, survival periods were recorded daily until mice reached their humane endpoint. As shown in Figure 6A, 1 × 10^3^ tachyzoites of the virulent RH strain significantly affected all animals across different mouse strains; however, mice immunized with pVAX-ROP5, pVAX-ROP18, pVAX-GRA7, pVAX-GRA15, or pVAX-MIC6 exhibited significantly longer survival times compared to the three control groups (*p* < 0.05). Co-administration of pVAX-IL-24 with pVAX-ROP5 + pVAX-ROP18 + pVAX-GRA7 + pVAX-GRA15 + pVAX-MIC6 resulted in the longest survival time compared to the three control groups (*p* < 0.05). In contrast, mice in the three control groups reached their humane endpoint within 6 to 21 days post-challenge (*p* > 0.05). As shown in Figure 6B, following DNA immunization with pVAX-IL-24 and the multi-gene combination, a lethal dose of 100 tissue cysts of the *T. gondii* ME49 strain induced only 10% mortality in BALB/c and Kunming mice and 40% mortality in C57BL/6 mice.

**Figure 7:**
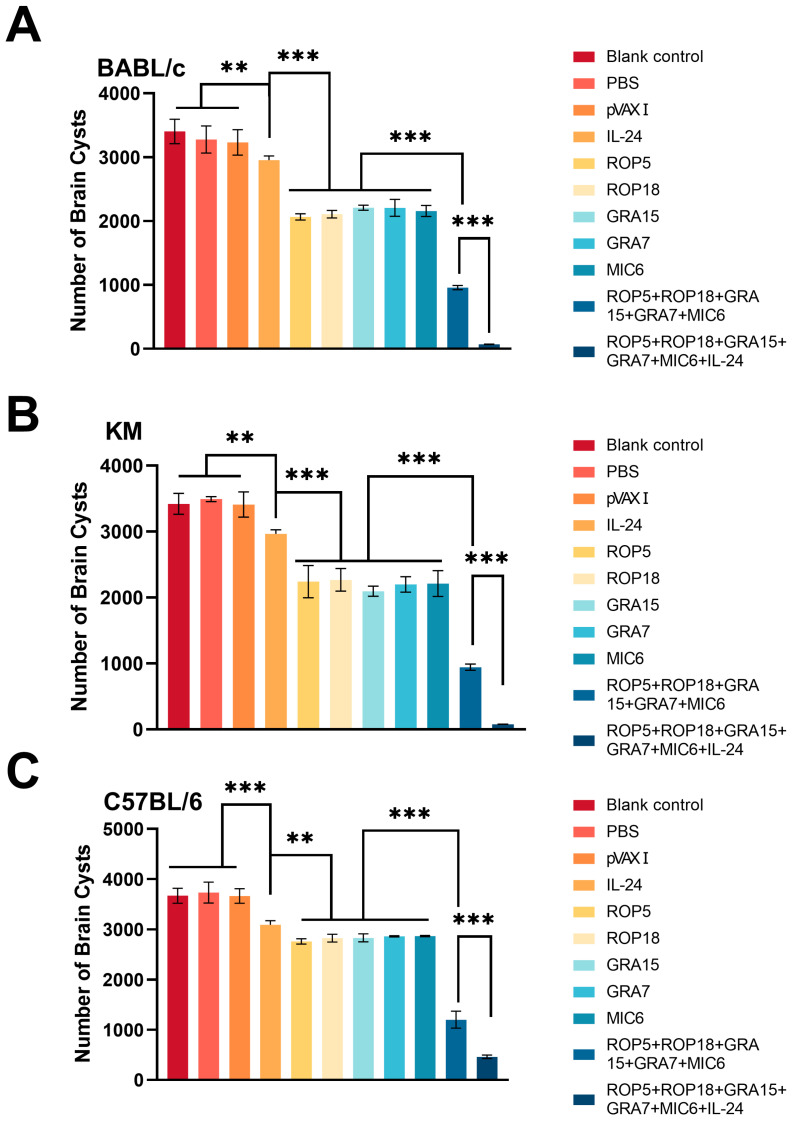
Protection against chronic toxoplasmosis in immunized mice two weeks after the final booster immunization. (**A**) Cyst reduction in immunized BALB/c mice, *n* = 6/group. (**B**) Cyst reduction in immunized C57BL/6 mice, *n* = 6/group. (**C**) Cyst reduction in immunized Kunming mice, *n* = 6/group. Bars represent the mean cyst burden per mouse brain following an oral challenge with 10 cysts of the ME49 strain. Cyst load was determined from whol-brain homogenates collected four weeks post-challenge. Data are presented as means ± SD (representative of three experiments). Statistical significance is indicated as *** *p* < 0.001, and ** *p* < 0.01 compared to control groups.

To evaluate the protective efficacy of the immunization regimen, vaccinated and control mice were challenged with a non-lethal dose of 10 tissue cysts from the *T. gondii* ME49 strain, and the mean number of cysts per brain was determined. Regarding cyst reduction across different mouse strains, as shown in Figure 7, the number of cysts in the brains of mice was significantly reduced in the pVAX-IL-24 + pVAX-ROP5 + pVAX-ROP18 + pVAX-GRA7 + pVAX-GRA15 + pVAX-MIC6 (98% in BALB/c, 97.5% in Kunming, 87.5% in C57BL/6) and pVAX-ROP5 + pVAX-ROP18 + pVAX-GRA7 + pVAX-GRA15 + pVAX-MIC6 groups (84.5% in BALB/c, 82.3% in Kunming, 76.8% in C57BL/6) compared to the control group (*p* < 0.05). However, no significant reduction in brain cyst numbers was observed among the three control groups (*p* > 0.05).

## 4. Discussion

The potential threat posed by *T. gondii* tissue cysts to both animal and public health underscores the urgent need for effective immunoprophylaxis strategies, especially given the lack of practical treatments for eliminating these cysts [16,17]. Despite significant progress in developing anti-*T. gondii* vaccines, there is a notable scarcity of research on the “best” antigens or optimal combinations specifically targeting *T. gondii* tissue cysts. Our study provides significant insights into the development of a multi-antigen DNA vaccine for *T. gondii*, demonstrating that immunization with a combination of TgROP5, TgROP18, TgGRA7, TgGRA15, and TgMIC6, along with the adjuvant cytokine IL-24, induces strong protective immunity against both acute and chronic toxoplasmosis. The robust humoral and cellular immune responses observed in our mouse models suggest that this vaccination strategy could serve as an effective prophylactic approach against *T. gondii* infection. Previous studies have highlighted the importance of DNA vaccines in eliciting protective immunity against toxoplasmosis, particularly through the induction of strong Th1 responses characterized by IFN-γ production. However, single-antigen DNA vaccines have generally yielded limited protective efficacy, necessitating the exploration of multi-antigen approaches [7,17]. Our findings align with studies that have shown enhanced immune protection when multiple antigens are combined, reinforcing the hypothesis that a polyepitope strategy may provide superior immunity against *T. gondii* [13,18]. The addition of IL-24 as an adjuvant further enhanced vaccine efficacy, a novel finding that distinguishes our study from previous vaccine research.

DNA vaccines have been constructed to elicit protective immunity against both acute and chronic *T. gondii* infections, as evidenced by various challenges using different *T. gondii* strains in animal models [7,19]. However, it is critical to consider the challenge dose of *T. gondii*, as insufficient immunity may fail to protect against high doses of the lethal RH strain, which results in shorter survival times. More reasonable challenge protocols typically involve administering 80–100 cysts of the low-virulence PRU strain to evaluate survival [13,16]. Our results indicate that co-administration of the five antigens along with the adjuvant cytokine results in nearly complete protective immunity against the *T. gondii* ME49 strain, while DNA immunization with the same components yields only partial protection against a challenge with 1 × 10^3^ tachyzoites of the virulent RH strain, resulting in extended but limited survival across all animal models.

Testing vaccine candidates in mouse models with varied genetic backgrounds is essential. In this study, we employed two inbred strains (C57BL/6 [H-2b] and BALB/c [H-2d]) and one outbred strain (Kunming [H-2d]), each characterized by distinct major histocompatibility haplotypes and varying susceptibility to *T. gondii*-induced morbidity and mortality [20]. The combination of the adjuvant cytokine with the five DNA vaccine candidates significantly reduced the number of tissue cysts in the brains of immunized mice compared to controls, although the protection level varied by mouse strain. The immunization resulted in nearly complete resistance to brain cyst formation in BALB/c and Kunming mice, while C57BL/6 mice exhibited a significant reduction of approximately 90%. Furthermore, co-administration of the multi-antigen vaccine and adjuvant provided near-complete protection in BALB/c and Kunming mice following a challenge with 100 cysts of the low-virulence ME49 strain, whereas C57BL/6 mice only experienced partial protection, surviving 6 to 36 days longer. No full protective immunity was observed against tachyzoites of the virulent RH strain across these strains. The significant reduction in brain cysts in BALB/c and Kunming mice and the extended survival observed in C57BL/6 mice suggest that our vaccine induces strain-dependent immunity. This is consistent with previous findings that indicate genetic background plays a crucial role in host susceptibility and immune response to *T. gondii* [20].

Humoral immunity plays a critical role in resistance to *T. gondii* infection, as antibodies regulate parasite phagocytosis, prevent invasion, and stimulate the classical complement pathway [21,22]. B cells are essential for the antibody-mediated protective effects induced by vaccination [22]. Our findings show that immunized mice generated high levels of anti-*T. gondii* IgG antibodies, contributing to protective efficacy against subsequent *T. gondii* tachyzoite infections and controlling the reactivation of cysts during chronic infection.

Th1-type cytokine-mediated immune responses are crucial for host resistance to *T. gondii* [23]. Following vaccination, specific Th1-type cytokines are induced, contributing to protective immunity against *T. gondii* challenges [17]. IFN-γ is the principal effector molecule of Th1 lymphocytes, required for host resistance during the early stages of infection through mechanisms including tryptophan degradation and the production of nitrogen oxides (NO) for *T. gondii* clearance [24,25]. The production of IL-12 is also essential for host resistance to *T. gondii* [26]. In particular, the IL-12p70 subunit is recognized as a key determinant of Th1 cell immune responses, while IL-12p40 promotes T cell proliferation during both acute and chronic stages of *T. gondii* infection [27]. Moreover, IL-2 is important for regulating the proliferation and activity of cytotoxic T lymphocytes (CTLs), which are crucial for resisting *T. gondii* infections [17]. Our study found that spleen cells from immunized mice significantly produced Th1-type cytokines, including IFN-γ, IL-2, IL-12p70, and IL-12p40, consistent with previous studies involving multi-antigen vaccines [12,13]. Additionally, increased levels of Th2-type cytokines such as IL-4 and IL-10 were detected in the immunized mice, differing from those immunized with DNA vaccines encoding individual antigens like TgROP1 [28] and TgROM5 [29]. IL-4 enhances IFN-γ production in the later stages of infection; however, its absence can lead to increased susceptibility to severe toxoplasmic encephalitis [30]. Conversely, IL-10 plays a role in inhibiting inflammation and preventing severe immunopathology induced by pro-inflammatory cytokines during *T. gondii* infection [31]. High levels of IL-10 have also been observed in mice immunized with *T. gondii* mutants, which help modulate pathological damage caused by Th1 cytokine induction [32,33]. The observed Th1 and Th2 responses are particularly relevant, as they contribute to both parasite clearance and the prevention of excessive immunopathology. The strong IFN-γ and IL-12p70 responses confirm the essential role of Th1 immunity in resistance against *T. gondii*, while the presence of IL-10 and IL-4 suggests a regulatory mechanism that limits immune-mediated damage, supporting findings from prior research [34,35].

Cytokines are crucial for regulating the immune system and maintaining physiological balance, influencing pathological conditions [35]. As a member of the IL-10 cytokine family, IL-24 is known to play an important role in immune-mediated inflammatory diseases [36]. IL-24s role in immune modulation is an emerging area of interest in infectious disease research. While it has been studied primarily in the context of cancer immunotherapy, its ability to regulate inflammatory responses and promote immune cell activation suggests broader applications [37,38,39]. Our study is the first to demonstrate IL-24s potential as an adjuvant in a *T. gondii* vaccine, highlighting its role in enhancing both CD8+ T cell responses and humoral immunity. Given that previous studies have focused on cytokines such as IL-12, IL-15, and IL-21 as vaccine adjuvants [40,41,42], our findings provide a new perspective on cytokine-based immunomodulation in anti-parasitic vaccines.

The ability of our DNA vaccine to induce strong and durable immune responses supports its potential as a viable strategy for controlling toxoplasmosis in both humans and animals. Given the lack of an effective human vaccine, our findings could pave the way for further development of DNA-based prophylactic and therapeutic vaccines for high-risk populations, such as immunocompromised individuals and pregnant women. Additionally, the demonstrated efficacy against brain cyst formation suggests that this strategy may be useful in preventing chronic toxoplasmosis, a key challenge in vaccine development. From a veterinary perspective, effective vaccines against *T. gondii* could significantly reduce transmission in livestock and companion animals, thereby decreasing the zoonotic risk for humans. The economic impact of toxoplasmosis in agriculture, particularly in sheep and pigs, underscores the need for vaccination strategies that are both safe and effective [43,44]. The incorporation of IL-24 in future vaccine formulations could be explored further to optimize immunogenicity without inducing excessive inflammation.

While our study provides promising results, several questions remain unanswered. Future research should investigate the long-term immunity conferred by this DNA vaccine, including memory T cell responses and antibody durability. Additional studies should also evaluate the safety profile of IL-24 as an adjuvant, particularly in terms of potential immune overactivation and autoimmunity. Another important direction is to test this vaccine in alternative animal models that more closely resemble human immune responses, such as non-human primates. Furthermore, future research should explore alternative delivery methods, such as nanoparticle-based or electroporation-enhanced DNA vaccines, to improve antigen uptake and immune stimulation. Finally, clinical trials will be necessary to assess the feasibility of translating this vaccine strategy into a practical solution for human and veterinary use. In this study, the addition of pVAX-IL-24 to the group immunized with TgROP5, TgROP18, TgGRA7, TgGRA15, and TgMIC6 enhanced protective immunity, along with increased humoral immune responses, lymphocyte proliferation, and heightened Th1-biased and CD8+ T cell responses. This led to improved protective efficacy against both acute and chronic *T. gondii* infections in mice. Consistent with our previous studies on IL-21 and IL-15, as well as the synergy of IL-7 and IL-15 [8,45], IL-24 augments the protective immunity induced by DNA vaccines. Moreover, the administration of pVAX-IL-24 alone elicited considerable non-specific protective immunity against *T. gondii*, suggesting its potential as an immunotherapeutic modulator for *T. gondii* vaccines and even a possibility that IL-24 stimulants could be used as potential adjuvant drugs to prevent parasite infection or their potential role in combination with the described DNA vaccines, depending on its critical role in the anti-tumor drugs [46,47]. However, it is essential to investigate the possibility of adverse effects, including increased immune sensitization, severe toxicity, autoimmunity, and various immune-mediated inflammatory diseases in future studies. Given the promising findings of IL-24 as an adjuvant, future investigations should focus on understanding the underlying mechanisms through which IL-24 enhances immune responses. This could include the use of IL-24 knockout models and pathway analysis to identify key signaling pathways involved in immune activation and cytokine modulation.

## 5. Conclusions

Our findings demonstrate that a multi-antigen DNA vaccine encoding TgROP5, TgROP18, TgMIC6, TgGRA7, and TgGRA15 elicits strong humoral and Th1-skewed immune responses, conferring significant protection against both acute and chronic *T. gondii* infections across multiple mouse strains. The inclusion of IL-24 as a genetic adjuvant further amplified protective immunity, highlighting its potential as an immune-enhancing component in vaccine strategies. These results provide valuable insights into DNA-based immunization approaches against *T. gondii* and offer a foundation for developing more effective vaccines against apicomplexan parasites, with implications for both veterinary and human health.

## Figures and Tables

**Table 1:** DNA vaccination regimens used in this study.

Group	Content	Volume	Administration
pVAX I plasmids expressing ROP5 + ROP18 + GRA7 + GRA15 + MIC6 + IL-24	17 μg/plasmid	100 μL	Intramuscular
pVAX I plasmids expressing ROP5 + ROP18 + GRA7 + GRA15 + MIC6	20 μg/plasmid	100 μL	Intramuscular
pVAX I	100 μg/plasmid	100 μL	Intramuscular
pVAX-ROP5	100 μg/plasmid	100 μL	Intramuscular
pVAX-ROP18	100 μg/plasmid	100 μL	Intramuscular
pVAX-GRA7	100 μg/plasmid	100 μL	Intramuscular
pVAX-GRA15	100 μg/plasmid	100 μL	Intramuscular
pVAX-MIC6	100 μg/plasmid	100 μL	Intramuscular
pVAX-IL-24	100 μg/plasmid	100 μL	Intramuscular
Phosphate-buffered saline	-	100 μL	Intramuscular
Blank control	-	-	-

## Data Availability

The original contributions presented in this study are included in the article. Further inquiries can be directed to the corresponding author.
